# Cost effectiveness of Iran national plasma contract fractionation program

**DOI:** 10.1186/2008-2231-20-63

**Published:** 2012-10-22

**Authors:** Abdol Majid Cheraghali

**Affiliations:** 1 High Institute for Research and Education on Transfusion Medicine, Iran Blood Transfusion Organization and Faculty of Medicine, University of Baqiyatallah Medical Sciences, Tehran, Iran

**Keywords:** Iran, Cost effectiveness, Plasma contract fractionation, Blood transfusion service

## Abstract

Plasma derived medicines (PDM) including immunoglobulins, clotting factors and albumin are life saving medicines which due to their high costs are inaccessible for many patients living in developing countries. By contrary substantial volume of plasma as raw materials for production of these medicines are discarded worldwide. Good quality recovered plasma, as a result of separation of donated blood into its components, could be used for production of PDM. In 2011 Iranian donors donated about 2 million units of blood. A shift from administration of whole blood to components therapy has resulted in the generation of over 250,000 liters of surplus of recovered plasma. This created a good opportunity for Iran’s health care system to use this plasma for production of PDM. Therefore Iran national transfusion service has started a contract fractionation program for converting recovered plasma into PDM. This program not only provided essential PDM for Iran pharmaceutical market but also has created a direct saving of about 8.5 million Euros in 2011 for national health sector. In addition this program has drastically contributed to improvement of overall quality of working procedures and services provided by Iran national blood transfusion organization.

## Background

Although blood as a very precious human resource has been used for direct transfusion for centuries it has recently become increasingly important to separate the manufacturing aspect of blood as a source of pharmaceutical “raw material”. The process of separating blood into its components will result to plasma as a rich source of human proteins for further manufacturing of plasma derived medicines (PDM). Fractionation of plasma could provide a wide range of medicines essential for management of patients with bleeding and immunological disorders. Coagulating factors including factor VIII (FVIII), factor IX (FIX), von willebrand factor, fibrinogen, fibrin sealants, prothrombin complex concentrate, albumin and immunoglobulins (IG) are among the most used PDM around the world. Some of these medicines including coagulation factors concentrates and IG are on the *WHO Model List of Essential Medicine* emphasizing their clinical importance. Primary immune deficiencies (PID) and bleeding disorders such as haemophilia are among the most important disease require PDM for their management. The majority of patients with haemophilia or PID are currently not receiving adequate treatment, and the numbers of these patients being diagnosed are increasing.

Global need for PDM have been increasing almost geometrically over the last decade and based on evidences for new indications of this medicines it seems that administration of PDM will increase for years to come [1]. Despite introduction of biotechnology for production of some of these medicines including clotting factors VIII and IX availability of others such as normal and hyperimmune IG and even albumin is limited only by the availability of plasma as their raw material for fractionation. It is estimated that every year about 30 million liters of plasma fractionated worldwide [2]. However, there are considerable discrepancies among countries in production and fractionation capacities for human plasma. Currently most plasma collection and fractionation capacity are located in North America, Europe and south East Asia. Although in some countries fractionation facility may rely on recovered plasma donated by voluntary whole blood donors, many others receive plasma produced through plasmapheresis from remunerated donors. Currently about 75% of plasma used for fractionation produced by plasmapheresis. Despite good availability of PDM in high resource countries many patients living in low resource countries in Africa, South and Central America, and Asia do not have timely access to PDM [2,3].

High costs and low availability of PDM in middle and low income economies resulted to under treatment of patients living in these countries. Based on data presented by patient oriented organizations [4,5] the majority of patients with clotting disorders, immune deficiencies, and autoimmune disorders living in middle and lower income economies do not have adequate access to PDM. Therefore availability of additional plasma for fractionation could be used to generate essential PDM and even national self sufficiency in plasma products can be achieved by reducing wastage of recovered plasma. According to the number of donated blood worldwide an estimated 21.6 million liters of plasma could be recovered from these donations. Based on number of plasma bags transfused for clinical use, it is estimated that at least 9.3 million liters of plasma are discarded annually [6,7].

Local fractionation of plasma and contract fractionation are two main options for using recovered plasma as an Active Pharmaceutical Ingredient (API) for production of PDM. Although both options will provide means to improve quality of the transfusion system and availability of PDM, local fractionation requires substantial investment of time and money due to the very high level of complexity of the plasma fractionation technology and regulatory issues [8]. However, in both cases availability of sufficient volume of plasma with acceptable quality is a pre requisite for starting any activity on fractionation of plasma. Unlike small molecule medicines cost related to the raw materials for producing PDM is the costliest part of the manufacturing procedure. Plasma represents approximately 30–40% of the cost of PDM [3]. Therefore any program intended to use produced plasma could create substantial saving on behalf of both national health service and patients. Countries which are able to produce plasma meeting quality requirements for fractionation in sufficient volume (e.g. from 10,000 to 30,000 liters) could establish a contract fractionation program. Contract fractionation program could provide a safe, secure and reliable supply of life-saving PDM [8].

In addition to other advantages of contract fractionation including tangible effects on quality of the blood transfusion system and transfusion safety [9] it could also serve as a good educational tool to build-up knowledge in the field before possible establishment of a domestic fractionation plant. Involvement of national regulatory authority in contract fractionation project would provide training field for local regulators to be prepared for regulation of possible future domestic fractionation facility.

However, success of any contract fractionation program requires some essential pre requisites. A contract fractionation agreement is a mutual activity that should follow transparent and well-established agreements between plasma supplier and fractionator company. Among other items volume and quality of plasma and costs of fractionation are of the utmost importance. Range of the products should be well-balanced based on the country’s needs to PDM. Therefore, successful contract fractionation programs could serve as a ramp-up phase for establishment of local fractionation facility, wherever feasible. That is why some developing countries with contract fractionation program have established a long term plan for establishment of local fractionation facility in their national policy for plasma. However, due to practical difficulties regarding transfer of plasma fractionation technology this major move should be done only after a very careful assessment and feasibility study based on presence of strong government endorsement and in particular the volume of plasma available. Since building a high tech GMP-compliant plasma fractionation facility require considerable investment, contract fractionation activity could be considered a valuable training activity for national staff to build a team of skilled manpower.

Despite presence of considerable number of fractionation facilities worldwide, there are only few countries which have reached full self sufficiently for providing PDM for their own market and most of the countries have to rely on importation of PDM in order to response to the patients’ need. Although more than 50% of global fractionators are located in the Asia-Pacific region, many fractionation plants in this region have small capacity or produce short range of the products. The total plasma volume fractionated in 2010 in this region is close to 6.4 million liters. The coverage of local needs by products prepared from domestic Japanese plasma is about 25% for factor VIII, 58% for albumin and 95% for IG in 2010. The volume of plasma fractionated in Korea is about 870,000 liters. China with over 25 plasma fractionation in 2010 fractionated about 3.4 million liters of plasma which has been collected exclusively by plasmapheresis from remunerated donors [2].

## Finding

### Iran contract fractionation program

Iran, a country with a population of over 74 millions, has one of the largest pharmaceutical market in the Middle East. Blood transfusion In Iran is an integral part of the national health system. In 1974 based on a parliamentary law, Iran Blood Transfusion Organization (IBTO) was established in order to centralize all blood transfusion activities from donor recruitment to production of blood components in one organization. IBTO is a public and non-profit organization that relies on the government of Iran for its budget and delivers all its basic services including blood components free of charge both to the public and private hospitals. Self sufficiently of country for blood and blood components has always been the main objective of IBTO. Since 2007 Iran has reached to 100% voluntary donation and IBTO collects blood only from non remunerated voluntary blood donors. In 2011 Iranian donors donated about 2 million units of blood to IBTO. In Iran in addition to the screening of the donors through interviewing of the donors all donated bloods undergo lab testing for possible presence of important and known blood borne diseases including hepatitis B virus (HBV), hepatitis c virus (HCV), human immunodeficiency virus (HIV) and syphilis. Percent of confirmed HBsAg positive samples in blood donated by Iranian donors has drastically decreased from 0.8% in 2002 to 0.25% in 2010. Prevalence of confirmed HCV positive results has also decreased from 0.18% in 2002 to 0.07% in 2010. Despite increasing prevalence of HIV in general population IBTO succeeded to reduce confirmed positive HIV samples in donated bloods to 0.004 in 2010 [10].

In recent decade administration of whole blood in health centers of Iran has drastically declined and therefore majority of donated blood (>95%) are now converted to blood components including packed red blood cells, platelets and plasma. A shift from administration of whole blood to component therapy has resulted in the generation of surplus of recovered plasma from donated blood. This created a good opportunity for Iran's health care system to use this plasma for production of PDM. In the other hand due to the improvement of standards of care, consumption of PDM in Iran shows a steady rise causing a significant burden on limited resources available in national health sector [9].

**Figure 1:**
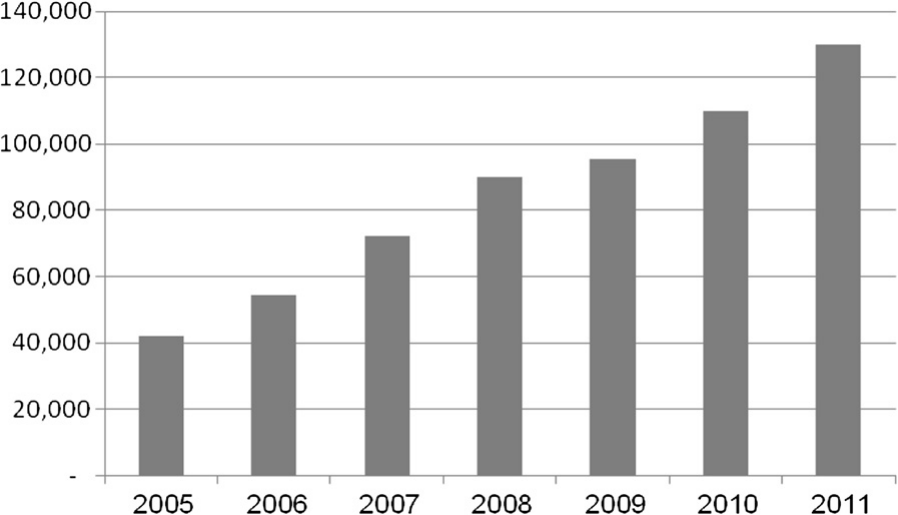
Volume of plasma (liters) shipped by IBRF for fractionation, 2005–2011.

Based on annual number of whole blood donation, IBTO is able to produce about 250,000 liters of surplus plasma annually. Therefore in order to use this plasma for improving availability and affordability of PDM and due to lack of any national fractionation facility, since 2005 IBTO and its sister company Iran Blood Research and Fractionation Co. (IBRF) initiated a contract fractionation program. Although improving the blood safety profile of the country through improving quality assurance system has been one of the main objectives of this project, the program has also significantly improved availability of PDM in the country [9]. IBTO has started contract fractionation program with about 40,000 liters of plasma. However, the volume of plasma shipped for fractionation has drastically increased in next years and reached to over 130,000 liters in 2011 (Figure 1). Although several countries both with developed and developing countries using contract fractionation as a means for providing PDM, few countries have reported economical impacts of their programs [11,12].

### Cost effectiveness evaluation

Obviously the medicines produced from this volume of plasma plays a critical role in providing essential PDM to Iranian patients. Plasma is a very valuable raw material and should be converted to as much medicines as practically possible. It has been reported that the cost of one liter of plasma, meeting the requirements for fractionation, is worth between 60–100 Euros [13]. Therefore, the production yield was one of the major considerations for IBRF when negotiating with fractionators. In addition in order to reach to a balanced product mix and optimize the cost effectiveness of plasma fractionation it is necessary that each liter of plasma should be used to produce as many PDM as possible. The average yields of products produced from one liter of recovered Iranian plasma in contract fractionation program are shown in Table 1. Average annual consumption of PDM in Iran market and share of PDM obtained through national contract fractionation program are also depicted in Table 2. In 2011 Iran national contract manufacturing project succeeded to produce 100% of IVIG and FIX, 15% of FVIII and 40% of albumin consumed in Iran’s market.

**Table 1:** Average yields of PDM/L of plasma

IVIG	4.5 g
FIX	200 IU
Albumin	24.5 g
FVIII	160 IU

PDM, Plasma derived medicines; IVIG, Intravenous immunoglobulin; FIX, Factor IX; FVIII, Factor VIII; IU, International unit.

**Table 2:** Annual average consumption of PDM in Iran and share of IBTO contact fractionation program in the market (2011)

**Medicine**	**Average annual consumption**	**% of Market share**
IVIG	700 kg	100
FVIII	120 MIU	15
FIX	15 MIU	100
Albumin	8,500 Kg	40

PDM, Plasma derived medicines; IVIG, Intravenous immunoglobulin; FIX, Factor IX; FVIII, Factor VIII; MIU, Million international unit.

In contrary to blood and blood components which are currently provided by IBTO free of charge for all Iranian patients, PDM in Iran are regarded as medicines and should be paid for. Except for antihemophilic factors VIII and IX which are highly subsidized by the government and are free of charge for hemophilia patients, cost of IG and albumin should be borne by either insurance companies or patient. IBTO and IBRF are non-profit organizations which have to recover their operational costs for contract fractionation including costs for plasma preparation from donated bloods, its transportation and fractionation costs through products provided. As expected, the price of PDM prepared by this program is still notably lower than those of commercially available in the market (Table 3).

**Table 3:** Differences between average prices of PDM produced through the contract fractionation program compared with market average for imported PDM (2010)

**Medicine**	**Average prices of PDM for contract fractionation program (€/unit)**	**Market average (€/unit)**
IVIG	29.5/g	40/g
Albumin	1.9/g	2.3/g
FVIII	0.20/IU	0.22/IU
FIX	0.21/IU	0.25/IU

PDM, Plasma derived medicines; IVIG, Intravenous immunoglobulin; FIX, Factor IX; FVIII, Factor VIII; IU, International unit.

Despite strong support of the activity by governmental officials especially Iran Ministry of Health, Iran contract fractionation program does not receive any financial support from the government and operates based on cost recovery approach. Therefore average prices of PDM produced through the national fractionation activity consists of contract fractionation costs, overhead and administration costs, operational costs and costs for running and expanding the program (e.g. construction of cold warehouses, procuring equipments, refrigerated trucks). IBRF has recently constructed a new 50,000 liters capacity cold warehouse devoted to the program. Based on data presented in Table 3 PDM produced through plasma fractionation activity is substantially less expensive compared to the commercially available medicines in the Iran market. This obviously created a direct savings for Iran public health budget as explained in Table 4. This program in 2011 has created about 8.5 Million Euros direct saving for Iran national health care system.

**Table 4:** Annual direct saving as a result of producing PDM through contract fractionation program compared with importing such medicines (2011)

**Medicine**	**Saving/unit (€)**	**Annual consumption**	**Annual saving (€)**
IVIG	9.5/g	650 kg	6,175,000
Albumin	0.4/g	8,500 kg	1,360,000 (based on 40% market share)
FIX	0.04/IU	15 MIU	600,000
FVIII	0.02/IU	120 MIU	420,000 (based on 15% market share)

PDM, Plasma derived medicines; IVIG, Intravenous immunoglobulin; FIX, Factor IX; FVIII, Factor VIII; MIU, Million international unit.

However, in addition to substantial direct effect of this project on saving of resources allocated for importation of PDM, indirect influence of the IBTO project on reducing costs of imported commercial medicines is worth mentioning. Availability of medicines produced from Iranian plasma in the market has inevitably forced the importers to offer more reasonable prices for their products in order to hold their shares in Iran’s market. In fact, the presence of the products produced from contract fractionation in the market, through improving bargaining power of the national authorities on commercial products, has contributed greatly to maintain a downward pressure on the price of the commercially available PDM in Iran’s market [12].

## Discussion

Although treatment of patients such as hemophilia and PID patients with PDM is a life saving intervention, in developing countries mainly due to limited resources available in their national health sector, most of these patients are not diagnosed or properly treated. Therefore use of PDM in these countries is much lower than the actual needs of patients [4,5]. Despite moderate increase in demand for clotting factors and albumin in recent years due to improved patient care and new clinical indication demand for IG therapy has greatly increased and is now the driver for plasma collection industry [1].

By improving standards of practice in transfusion medicine many countries even in developing countries turned to components therapy instead of administration of whole blood. Therefore as a consequence, millions of additional plasma units could be generated which unfortunately under current conditions are discarded. Wastage of plasma in these countries is not only could be considered as a human tragedy but also is an economic misfortune for their heath sector. Based on number of donated blood globally it is estimated that every year at least 9 million liters of recovered plasma discarded worldwide. Therefore based on current prices for one liter of plasma such wasted plasma has a market value of about 650–900 million USD. Use of this volume of plasma theoretically could result in about 1.4 billion International Unit (IU) of FVIII. Based on annual use of 20,000 IU per patient this quantity of FVIII would allow an additional 70,000 children with haemophilia A to be treated each year. In addition, the same volume of plasma can generate an additional 2.3 billion IU of FIX necessary for treatment of an additional 57,500 haemophilia B children each year, assuming an annual consumption of 40,000 IU/year/patient. More importantly the same volume of recovered plasma could produce at least 37 tons of IG. This quantity would be used to manage hundred thousand of PID patients. Finally, 230 tons of albumin calculated on a mean recovery of 25 g/L, could be produced from this volume of plasma. This quantity would cover the needs of billion inhabitants based on a consumption of 200 kg/million population. It should also be kept in mind that plasma can be fractionated to yield other PDM as well.

Therefore it is both ethical and economically feasible for these countries to take measures to preserve and use this plasma as API for production of PDM. Recovering this plasma and turning it into essential medicines would meet a substantial unmet health need. Additionally such program will eventually improve national capability to provide all blood components of higher quality and safety through upgrading blood collecting facilities to the status of pharmaceutical manufacturers with acceptable GMP [9]. Currently in developing countries much of the plasma generated as a side product of component preparation due to its low quality and absence of appropriate and standard freezing or storage capacity is unusable for fractionation and therefore discarded. This could not only be considered as wastage of valuable resources but also may create environmental contamination. Obviously improving GMP in blood establishments in these countries will conserve such plasma.

Contract fractionation of locally produced plasma could substantially improve affordability of PDM especially in developing countries. In addition to saving on the budget a contract fractionation program could also protect the country from product shortages. In majority of cases the contract fractionation program will provide a reliable and affordable supply of PDM. However, it should be mentioned that contact fractionation could attract hostility from importers of PDM and the program can be exposed to competition from temporarily low prices for PDM in national market as result of “dumping” by the importers. Therefore, contract fractionation programs should be considered as a long term activity and should not be reconsidered based on short term fluctuation of PDM prices in the market.

## Conclusion

According to the WHO Secretariat Report in 2010 “a large percentage of the plasma collected in developing countries is categorized as waste material and destroyed. This wastage occurs because appropriate technology, regulatory controls, quality systems and good manufacturing practices are all lacking, thereby rendering the plasma unsuitable for conversion into fractionated medicinal” [7]. Therefore by improving quality of working procedures a national blood service that separate plasma from donated blood could supply it to a fractionator in exchange of PDM in a plasma contract fractionation program or, when justified, local production. In addition, contract manufacturing programs could be considered as a means to improve the transfusion system and ensure the availability of safe blood products. In order to be able to use recovered plasma as API each unit of recovered plasma should meet quality and safety requirements. This is a fundamental pre-requisite to any fractionation program.

It is obvious that successful fractionation programs require commitment and investment to establish, both on the part of the blood collection service and the contract fractionator. In any cases success of contract fractionation activity requires strong and reliable governmental commitment, effective framework, balanced products basket and long term planning.

In order to be viable any contract fractionator program needs to recover the establishment costs. Well planned contract fractionation programs which rely on large enough volume of plasma is a cost effective approach. Iran, as a country faced with increasing demand for PDM relies on its centralized blood transfusion system for producing plasma for fractionation and has successfully implemented this approach in past years to improve availability and affordability of these medicines. Iran contract fractionation program has enjoyed strong government commitment and support which should be considered as one the main success elements of this program. Iran experience has shown that contract plasma fractionation through direct and indirect effects on price of PDM could substantially improve availability and affordability of such medicines in national health market. In conclusion it is obvious that there is a need for increased global plasma collection and fractionation into PDM and enhanced regulatory capacity in developing countries to make the best use of recovered plasma. Therefore, contract fractionation projects for the production of PDM remain a practical approach to respond to the needs of patients to PDM.

## Competing interests

The author declare that he/she has no competing of interests.

## Author information

Professor Abdol Majid Cheraghali is advisor to Managing Director of IBTO, board member of IBRF and a key person for Iran national plasma contract fractionation program.
